# Distinguishing Hepatocellular Carcinoma From Hepatic Inflammatory Pseudotumor Using a Nomogram Based on Contrast-Enhanced Ultrasound

**DOI:** 10.3389/fonc.2021.737099

**Published:** 2021-10-07

**Authors:** Mengting Liao, Chenshan Wang, Bo Zhang, Qin Jiang, Juan Liu, Jintang Liao

**Affiliations:** ^1^ Department of Ultrasonography, Xiangya Hospital, Central South University, Changsha, China; ^2^ Health Management Center, Xiangya Hospital, Central South University, Changsha, China; ^3^ Department of Medical Ultrasound, Wuhan First Hospital, Wuhan, China

**Keywords:** contrast-enhanced ultrasound (CEUS), inflammatory pseudotumor (IPT), hepatocellular carcinoma (HCC), nomogram, LASSO regression

## Abstract

**Background:**

Hepatocellular carcinoma (HCC) and hepatic iflammatory pseudotumor (IPT) share similar symptoms and imaging features, which makes it challenging to distinguish from each other in clinical practice. This study aims to develop a predictive model based on contrast-enhanced ultrasound (CEUS) and clinical features to discriminate HCC from IPT.

**Methods:**

Sixty-two IPT and 146 HCC patients were enrolled in this study, where pathological diagnosis served as the reference standard for diagnosis. Clinical and ultrasound imaging data including CEUS features: enhancement degree during arterial phase, portal phase and delayed phase, enhancement pattern, early washout within 60 s, feeding artery, peritumoral vessels, peritumoral enhancement, and margin of nonenhanced area were retrospectively collected. Imaging data were reviewed by two experienced ultrasound doctors. Patients were randomly assigned to training and validation sets. Chi-squared test followed by LASSO regression was performed on ultrasonographic features in the training set to identify the most valuable features that distinguish HCC from IPT, based on which the sonographic score formula was generated. With the significant clinical and ultrasonographic indicators, a nomogram was developed. The performance of the nomogram was verified by ROC curve and decision curve analysis (DCA) with the comparison with sonographic score and the ultrasound doctor’s diagnosis.

**Results:**

The most valuable ultrasonographic features that distinguish between HCC and IPT were enhancement degree during arterial phase, early washout, peritumoral vessels, peritumoral enhancement, and liver background. The sonographic score based on these features was verified to be an independent factor that predicts the diagnosis (*p* = 0.003). Among the clinical indicators, AFP (*p* = 0.009) and viral hepatitis infection (*p* = 0.004) were significant. Sonographic score, AFP, and viral hepatitis were used to construct a predictive nomogram. The AUC of the nomogram was 0.989 and 0.984 in training and validation sets, respectively, which were higher than those of sonographic score alone (0.938 and 0.958) or the ultrasound doctor’s diagnosis (0.794 and 0.832). DCA showed the nomogram provided the greatest clinical usefulness.

**Conclusion:**

A predictive nomogram based on a sonographic signature improved the diagnostic performance in distinguishing HCC and IPT, which may help with individualized diagnosis and treatment in clinical practice.

## Introduction

Inflammatory pseudotumor (IPT), synonymous with inflammatory myofibroblastic tumor, is an uncommon benign neoplasm characterized by spindle cell infiltration and various degrees of inflammatory cells (1). Hepatic IPT often presents with atypical symptoms such as abdominal pain, fever, and nonspecific lab results, and therefore diagnosis is challenging and largely relies on the pathologic findings (2, 3). Moreover, IPT has a potential for mimicking malignant tumors, such as hepatocellular carcinoma (HCC) (4–6). Around 60% of IPT patients had to receive surgery because a malignancy was suspected even with the imaging results (3).

HCC is an aggressive cancer and the third leading cause of cancer death worldwide (7). In China, hepatitis virus infection serves as a major risk factor which leads to cirrhosis and ultimately HCC (8). Unfortunately, diagnosis of HCC is often made at advanced stages, and 5-year survival rate is less than 15% (9, 10). Early detection of HCC depends not only on screening biomarkers such as alpha fetoprotein (AFP) but also imaging examinations. Contrast-enhanced ultrasound (CEUS) is one of the most common noninvasive diagnostic methods (11).

However, HCC shares some overlapping ultrasonographic features with IPT (1, 3, 6). Typical CEUS for HCC is a quick wash-in in the arterial phase followed by a quick wash-out (12). Sustained enhancement in the late phases can be observed in well-differentiated HCC cases (13, 14). On the other hand, IPT as a benign tumor, have no enhancement in CEUS. However, atypical IPT presents with various patterns of enhancement in the arterial phase with hypoenhancement during the portal and delayed phases, and 40% of them had quick wash-in and wash-out, which resembles the CEUS feature of HCC (3, 6). Additionally, HCC in two-dimensional ultrasound is hypoechoic, and dark areas of fluid or heterogeneous echo can appear when necrosis or hemorrhage occurs (11). Meanwhile, IPTs can also be heterogeneous and mixed with echogenic and anechoic compartments, resembling the characteristics of HCC (1). Therefore, to distinguish HCC from IPT can be difficult, but the preoperative diagnosis is crucial for clinical assessment and decision making. To develop a predictive system that helps with differential diagnosis would be beneficial in clinical practice.

To the best of our knowledge, there has been no study that develops a model to distinguish between HCC and IPT. Due to the rareness of IPT, most literatures described IPT in case reports (5, 6). In our study, a large cohort tracked over a period of 10 years was analyzed. By taking the advantage of bioinformatics analysis, the most differential clinical and ultrasonographic features between HCC and IPT were identified, and an ultrasound-based nomogram model was constructed to distinguish HCC from IPT. The area under the curve (AUC) of the model was up to 0.98. With this study, we hope to improve the diagnostic performance for the benign IPT and malignant HCC, thus helping with better decision making for doctors in clinical practice.

## Material and Methods

### Study Population

**Figure 1:**
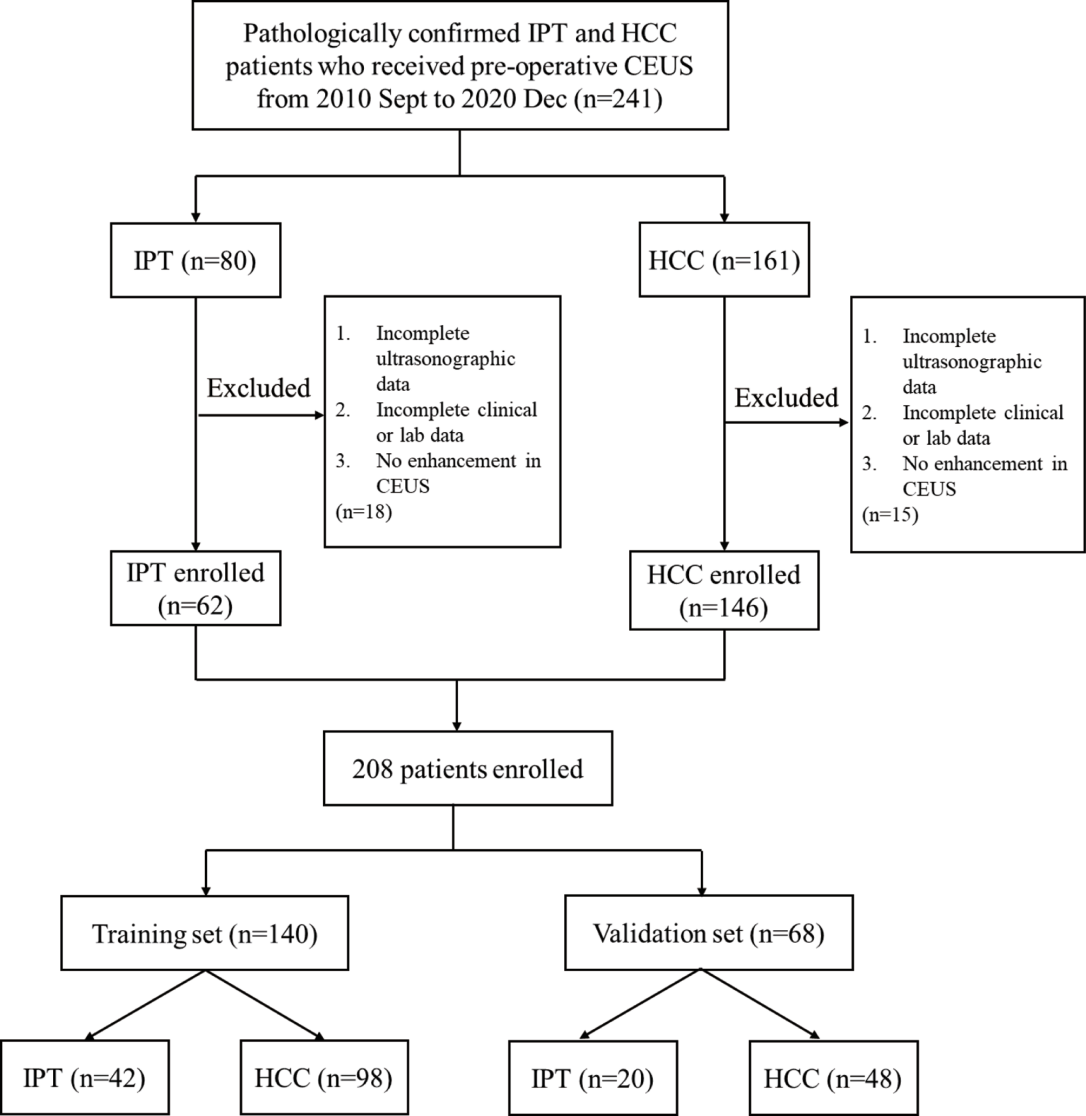
Flow chart of the study population selection.

The study was approved by the ethics committee of Xiangya Hospital Central South University. Patients who received CEUS followed by surgery or biopsy with pathologically confirmed diagnosis of IPT in Central South University Xiangya Hospital consecutive from 2010 September to 2020 December were included. Confirmed HCC patients consecutive from 2018 June to 2019 December were included as well. The inclusion criteria were as follows: (1) HCC: Pathologically confirmed HCC patients without previous chemotherapy or radiotherapy. (2) IPT: Liver pathological results showed inflammatory cell infiltration and fibrous hyperplasia, with no tumor cells observed, and there was enhancement in CEUS. The exclusion criteria were as follows: (1) Incomplete ultrasonographic data. (2) Incomplete epidemiological data or lab results. (3) There was no enhancement during any of the three phases in CEUS. The clinic-pathological data and ultrasonographic data were collected for the enrolled participants. We divided the final enrolled patients into training set and validation set with a ratio of 2:1 according to random generation number method. The flow chart of the study population selection process was shown in **Figure 1**.

### Clinicopathologic Information

Demographic information including age, sex, clinical information including symptoms (abdominal pain, fever, abdominal sign), lab test results including viral hepatitis (HBsAg or HCV-Ab positive), alpha-fetoprotein (AFP), CA199, CA125, white blood cell count (WBC), albumin and direct bilirubin (DBIL) were collected.

Pathological results were collected and served as the reference standard in this study. Briefly, biopsy or surgical specimens from the hepatic lesion were fixed with 10% formalin. Hematoxylin-eosin staining was performed on the tissue slices. The diagnosis was made by at least two experienced pathologists, and immunohistochemical and special staining methods were used if it is necessary for diagnosis.

### Ultrasound Examination

Ultrasound (US) examination was performed by one of the skilled ultrasound doctors with over 5 years of experience of liver CEUS. Baseline ultrasound and color Doppler flow imaging (CDFI) were performed, and the entire liver was fully scanned to obtain two-dimensional ultrasonic features. The equipment was then changed to contrast imaging mode, and a volume of 2.4 ml of SonoVue was intravenously injected to the patient *via* the antecubital vein. Dynamic contrast enhancement was recorded immediately. The arterial phase was 10–30 s after contrast agent injection, the portal venous phase was from 25 to 45 s until 120 s, and the delayed phase was 120–300s. Under circumstances where multiple nodules were observed or contrast effect was not satisfying, a second round of contrast examination would be carried out 10 min later.

The US equipment and settings were as follows: Philips IU22 (C5-2 transducer, frequency range 2.0–4.5 MHz), Siemens Acuson S2000 (4C1 transducer, frequency range 2.0–4.5 MHz), Hitachi-ARIETTA-70 (C251 transducer, frequency range 2.0–5.0 MHz), LOGIQ E9 (C1-6 transducer, frequency range 1.0–6.0 MHz). All instruments above were equipped with contrast imaging mode. Second-generation contrast agent sulfur hexafluoride microbubble SonoVue (Bracco, Italy) was used in contrast-enhanced ultrasonography.

### Ultrasonographic Data Collection

Ultrasonographic records were reviewed by two experienced ultrasound doctors (CW and BZ) separately to extract ultrasonographic features. When a diverse conclusion was made, a third senior doctor (JL) was consulted to get a consistent conclusion.

The two-dimensional ultrasonic features included: nodule location (left liver, right liver, junction of left and right liver, or caudate lobe), size, number (single or multiple), shape (regular or irregular), boundary (well-defined or poorly defined), echo distribution (homogeneous or heterogeneous), presence of anechoic area, internal echo (hypo, iso, hyper, heterogeneous, or mixed) and liver background (whether there was liver cirrhosis or diffuse parenchymal liver disease). In cases of multiple nodules, the one with the maximum diameter was considered the target lesion. The blood flow was graded into four types (grades 0, I, II, and III) based on the abundance according to Adler classification method (15). CEUS features were recorded as follows: enhancement during arterial phase, portal phase and delayed phase (hypo, iso, or hyper), enhancement pattern (homogeneous, heterogeneous, with distinct nonenhanced area, or ring enhancement), early washout within 60 s, feeding artery, peritumoral vessels, peritumoral enhancement, and margin of nonenhanced area (well-defined, poorly defined, or nonenhanced area absent).

### Sonographic Scoring Through Least Absolute Shrinkage and Selection Operator Regression

To screen the valuable ultrasonic features for differentiation of IPT and HCC, Chi-squared test was performed between IPT and HCC patients in the training set. With the significantly different features (*p* < 0.05), least absolute shrinkage and selection operator (LASSO) regression was performed to minimize the multicollinearity of the ultrasonic features. Coefficient of each variable was generated. To identify the most useful independent features to distinguish between HCC and IPT, the five ultrasonic features with the highest absolute value of coefficient were used to generate the formula for calculating sonographic score. Sonographic score = *β*_1_ * *X*_1_ + *β*_2_ * *X*_2_ + … + *β_n_* * *X_n_*, where *X* is the value for the ultrasonic feature, *β* is the coefficient for the feature, and *n* is the number of total ultrasonic features.

Based on the formula, sonographic score was calculated for each patient. To validate the performance of the scoring formula, a heatmap was generated to visualize the sonographic score for both training and validation sets. The sonographic score was compared with the pathological diagnosis to verify the consistency.

### Development and Validation of Nomogram to Distinguish between HCC and IPT

With the demographic, clinical features and sonographic score, univariate logistic regression was performed in the training set. With the variables with p<0.01 in univariate regression, multivariate logistic regression was performed. Variables with p<0.05 were considered as independent factors to differentiate HCC from IPT. A nomogram was constructed with the independent factors above to discriminate HCC from IPT.

To assess the effectiveness of the nomogram, we compared the performance of 3 models: (1) nomogram, (2) sonographic score and (3) the diagnosis made by the ultrasound doctor. Receiver operator characteristic (ROC) curves were generated for 3 models in both training set and validation set, and area under the curves (AUC) were calculated. Decision curve analysis (DCA) was conducted by quantifying the net benefits at different threshold probabilities, to evaluate the clinical usefulness of 3 models above in both training and validation sets.

### Statistical Analysis

Statistical analysis was conducted with IBM SPSS Statistics 23.0 and R software (version 4.0.4). Categorical variables were compared using the *χ* ^2^ test, and continuous variables were compared using the *t*-test for variables with a normal distribution or the Mann-Whitney *U* test for variables with an abnormal or unknown distribution. Univariate and multivariate regression analyses were conducted using bivariate logistic regression model. The statistical significance levels were two-sided, and *p* < 0.05 was considered statistically significant unless otherwise specified. Graphpad Prism 8 was applied to generate heatmap for sonographic score and compare with pathological diagnosis. In R software, “glmnet” package was used for LASSO regression, “forestplot” package was used to display the results of univariate and multivariate logistic regression analyses, and “rms” package was used to plot the nomogram. ROC curves were generated with SPSS. AUC was calculated and compared between models by “pROC” package in R. DCA was conducted using “rmda” package in R.

## Results

### Patients

Flowchart of patient selection is shown in **Figure 1**. According to inclusion and exclusion critera, a total of 208 patients (62 IPT and 146 HCC) were finally enrolled in this study. All 146 HCC patients were diagnosed by postoperative pathology. Of 62 IPT patients, 31 were diagnosed by percutaneous liver biopsy and 31 were diagnosed by surgery. There were 140 patients in the training set (105 males and 35 females, age 52.7 ± 13.1 years), consisting of 42 with IPT and 98 with HCC. There were 68 patients in the validation set (56 males and 12 females, age 54.6 ± 11.6 years), consisting of 20 with IPT and 48 with HCC.

Clinicopathologic characteristics and sonographic score are summarized and compared between training and validation sets in **Table 1**. There was no significant difference between two sets of patients, which justified the grouping of training and validation sets.

**Table 1:** Clinicopathologic characteristics of patients in the training and validation sets.

Characteristics (*n*, % or mean ± SD)	Training set (*n* = 140)	Validation set (*n* = 68)	*p*-Value
Sex			0.234
Male	105 (75.0)	56 (82.4)	
Female	35 (25.0)	12 (17.6)	
Age (years)	52.7 ± 13.1	54.6 ± 11.6	0.290
check (cm)	4.8 ± 3.2	4.8 ± 2.5	0.915
Nodule number			
Single	118 (84.3)	59 (86.8)	0.638
Multiple	22 (15.7))	9 (13.2)	
Nodule location			0.360
Left liver	42 (30.0)	15 (22.1)	
Right liver	94 (67.1)	53 (77.9)	
Junction of left and right liver	2 (1.4)	0 (0)	
Caudate lobe	2 (1.4)	0 (0)	
Abdominal pain			0.967
Yes	49 (35.0)	24 (35.3)	
No	91 (65.0)	44 (64.7)	
Abdominal signs			0.235
Yes	18 (12.9)	5 (7.4)	
No	122 (87.1))	63 (92.6)	
Fever			0.083
Yes	10 (7.1)	10 (14.7)	
No	130 (92.9))	58 (85.3)	
Viral hepatitis			0.132
Yes	86 (61.4)	54 (79.4)	
No	49 (35.0)	19 (27.9)	
AFP (µg/L)			0.212
≥20	60 (42.9)	23 (33.8)	
<20	80 (57.1)	45 (66.2)	
CA199 (kU/L)			0.153
≥35	10 (7.1)	9 (13.2)	
<35	130 (92.9)	59 (86.8)	
CA125 (U/ml)			0.354
≥35	6 (4.3)	5 (7.4)	
<35	134 (95.7)	63 (92.6)	
WBC (10^9/L)			0.259
≥9.5	25 (17.9)	8 (11.8)	
<9.5	115 (82.1)	60 (88.2)	
Albumin (g/L)			0.855
<40	76 (54.3)	36 (52.9)	
40–55	64 (45.7)	32 (47.1)	
Direct bilirubin (µmol/L)			0.572
≥6.8	56 (40.0)	30 (44.1)	
<6.8	84 (60.0)	38 (55.9)	
Radiomics score	2.72 ± 2.74	2.10 ± 1.76	0.089

### Identification of Ultrasonographic Features to Distinguish Between HCC and IPT

**Figure 2:**
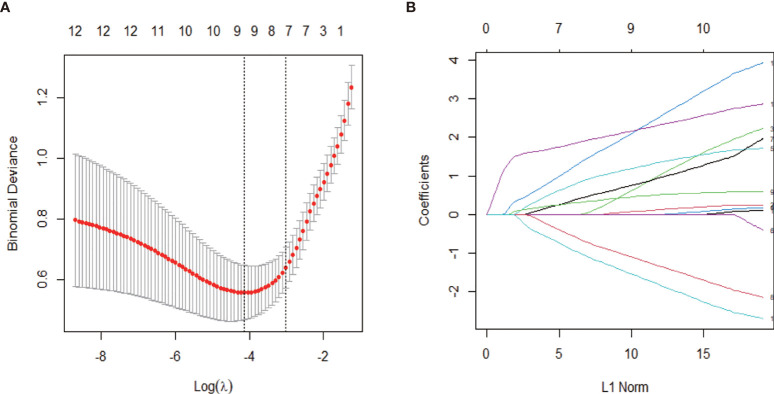
Ultrasonographic feature selection with least absolute shrinkage and selection operator (LASSO) regression in the training set. **(A)** The value of *λ* that gave the minimum average binomial deviance was used to select features. Dotted vertical lines were drawn at the optimal values using the minimum criteria and the 1-SE criteria. The optimal value of 0.049 was selected. **(B)** Coefficient profiles of the 12 ultrasonographic features.

Representative images of IPT and HCC are shown in **Supplementary Figures S1–S3**. To screen the ultrasonographic features that discriminate HCC and IPT, Chi-squared tests were performed on all the ultrasonographic features in training set. Twelve out of 16 features were significantly different (shown in **Table 2**): shape (*p* = 0.042), presence of anechoic area (*p* = 0.003), internal echo (*p* = 0.003), liver background (*p* < 0.001), CDFI (*p* = 0.041), arterial phase enhancement degree, (p<0.001), enhancement pattern (*p* = 0.001), early washout (*p* = 0.003), feeding artery (*p* < 0.001), peritumoral vessels (*p* < 0.001), peritumoral enhancement (*p* < 0.001), margin of nonenhanced area (*p* < 0.001). Representative images of feeding artery and peritumoral vessels are shown in **Supplementary Figures S3C, D**. With these ultrasonographic features, LASSO regression was applied to solve the multicollinearity relationships in those features, shown in **Figure 2**. Coefficient of each variable was generated.

**Table 2:** Comparison of ultrasonographic features between IPT and HCC in the training set.

Ultrasonographic features (*n*, %)	IPT (*n* = 42)	HCC (*n* = 98)	*p*-Value
Shape			0.042*
Regular	25 (59.5)	40 (40.8)	
Irregular	17 (40.5)	58 (59.2)	
Boundary			0.175
Well-defined	31 (73.8)	82 (83.7)	
Poorly defined	11 (26.2)	16 (16.3)	
Echo distribution			0.405
Homogeneous	14 (33.3)	40 (40.8)	
Heterogeneous	28 (66.7)	58 (59.2)	
Presence of anechoic area			0.003*
Yes	10 (23.8)	6 (6.1)	
No	32 (76.2)	92 (93.9)	
Internal echo			0.003*
Hypo	29 (69.0)	45 (45.9)	
Iso	1 (2.4)	4 (4.1)	
Hyper	2 (4.8)	10 (10.2)	
Heterogeneous	6 (14.3)	38 (38.8)	
Mixed	4 (9.5)	1 (1.0)	
Liver background			<0.001*
Liver cirrhosis or diffuse parenchymal liver disease	6 (14.3)	81 (82.7)	
Others	36 (85.7)	17 (17.3)	
CDFI			0.041*
Grade 0	21 (50.0)	32 (32.7)	
Grade I	8 (19.0)	11 (11.2)	
Grade II	10 (23.8)	34 (34.7)	
Grade III	3 (7.1)	21 (21.4)	
Arterial phase enhancement degree			<0.001*
Hypo	3 (7.1)	0 (0.0)	
Iso	10 (23.8)	2 (2.0)	
Hyper	29 (69.0)	96 (98.0)	
Portal phase enhancement degree			0.256
Hypo	27 (64.3)	49 (50.0)	
Iso	13 (31.0)	45 (45.9)	
Hyper	2 (4.8)	4 (4.1)	
Delayed phase enhancement degree			0.110
Hypo	33 (78.6)	89 (90.8)	
Iso	8 (19.0)	7 (7.1)	
Hyper	1 (2.4)	2 (2.0)	
Enhancement pattern			0.001*
Homogeneous	17 (40.5)	53 (54.1)	
Heterogeneous	3 (7.1)	12 (12.2)	
With distinct nonenhanced area	16 (38.1)	33 (33.7)	
Ring enhancement	6 (14.3)	0 (0)	
Early washout (<60 s)			0.003*
Yes	26 (61.9)	34 (34.7)	
No	16 (38.1)	64 (65.3)	
Feeding artery			<0.001*
Yes	18 (42.9)	84 (85.7)	
No	24 (57.1)	14 (14.3)	
Peritumoral vessels			<0.001*
Yes	13 (31.0)	75 (76.5)	
No	29 (69.0)	23 (23.5)	
Peritumoral enhancement			<0.001*
Yes	16 (38.1)	9 (9.2)	
No	26 (61.9)	89 (90.8)	
Margin of nonenhanced area			<0.001*
Well-defined	17 (40.5)	7 (7.1)	
Poorly defined	5 (11.9)	26 (26.5)	
Nonenhanced area absent	20 (47.6)	65 (66.3)	

*P < 0.05 was regarded as statistically signifcant.

### Development of Sonographic Score

In order to construct an effective sonographic formula with limited variables, the top 5 variables with the largest coefficient absolute value were chosen: arterial phase enhancement degree, early washout, peritumoral vessels, peritumoral enhancement, and liver background. Combined with the corresponding coefficients, the sonographic score formula was as follows: Sonographic score = 1.20026601 × arterial phase enhancement degree − 1.11632710 × early washout (<60 s) + 2.12170679 × peritumoral vessels − 1.55731319 × peritumoral enhancement + 2.17312021 × liver background.

**Figure 3:**
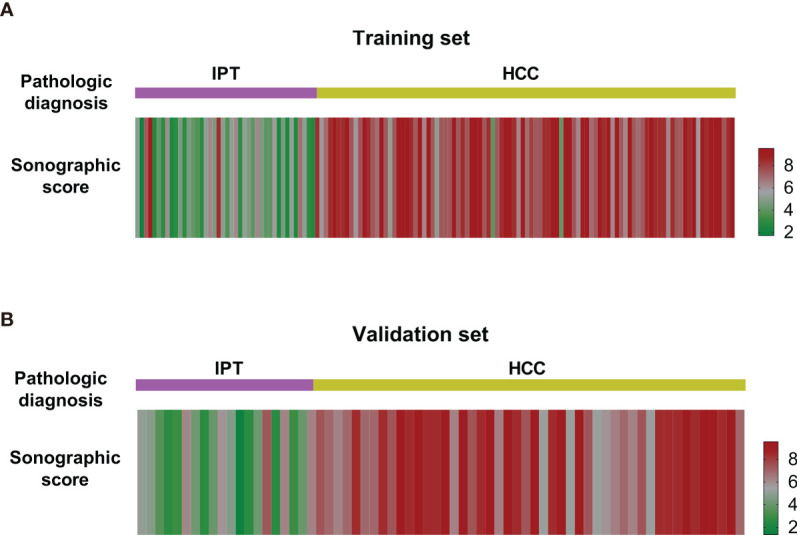
Heatmap of sonographic scores of IPT and HCC patients in the training set **(A)** and the validation set **(B)**. The scores of IPT and HCC patients were notably different from each other and consistent with the pathological diagnosis.

Sonographic score for all patients in training and validation sets were calculated according to the formula above. Heatmaps of the scores for IPT and HCC are shown in **Figure 3**. Patients with IPT and HCC had remarkably different sonographic scores, which were highly consistent with the pathological diagnosis.

### Screening Clinicopathologic Features

**Figure 4:**
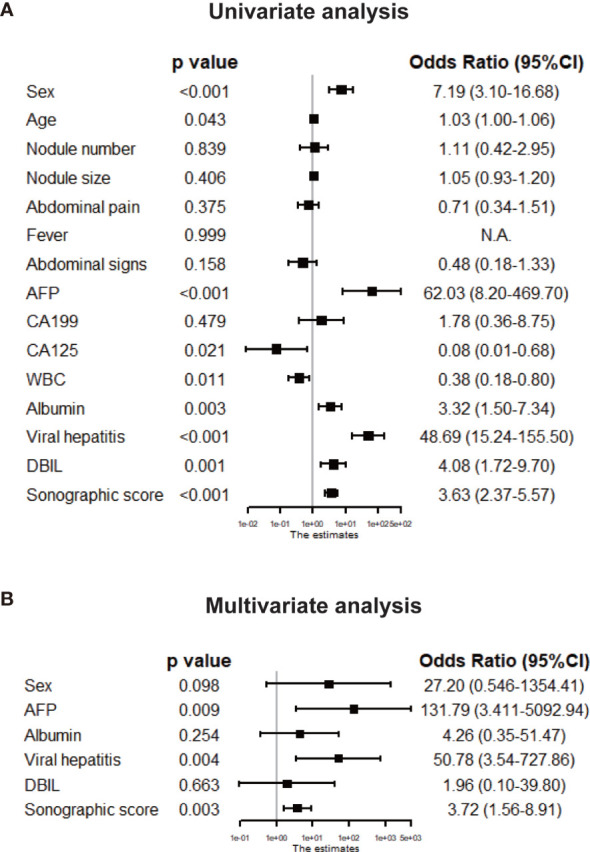
Tree diagram showing the univariate **(A)** and multivariate **(B)** logistic analyses of clinical features and sonographic score in the training set. The parameters with *p* < 0.01 in the univariate analysis were chosen for the multivariate analysis, and the features with *p* < 0.05 in the multivariate analysis were considered independent risk factors for HCC diagnosis.

Clinicopathologic features as well as sonographic score were compared between HCC and IPT in training set by univariate logistic regression and displayed in forest plot in **Figure 4A**. Sex [OR: 7.19 (95% CI 3.10, 16.68)], AFP [OR: 62.03 (95% CI 8.20, 469.70)], albumin [OR: 3.32 (95%CI 1.50, 7.34)], viral hepatitis (HBsAg or HCV-Ab positive) [OR: 48.69 (95% CI 1.50, 7.34)], DBIL [OR: 4.08 (95% CI 1.72, 9.70)] and sonographic score [OR: 3.63 (95% CI 2.37, 5.57)] were significantly different between HCC and IPT with *p* < 0.01. With these features, multivariate regression was performed to further identify independent factors. As shown in **Figure 4B**, AFP [OR: 131.79 (95% CI 3.41, 5092.94), *p* = 0.009], viral hepatitis [OR: 50.78 (95% CI 3.54, 727.86), *p* = 0.004] and sonographic score [OR: 3.72 (95% CI 1.56, 8.91), *p* = 0.003] were independent factors that distinguish HCC and IPT.

### Construction and Validation of Nomogram

**Figure 5:**
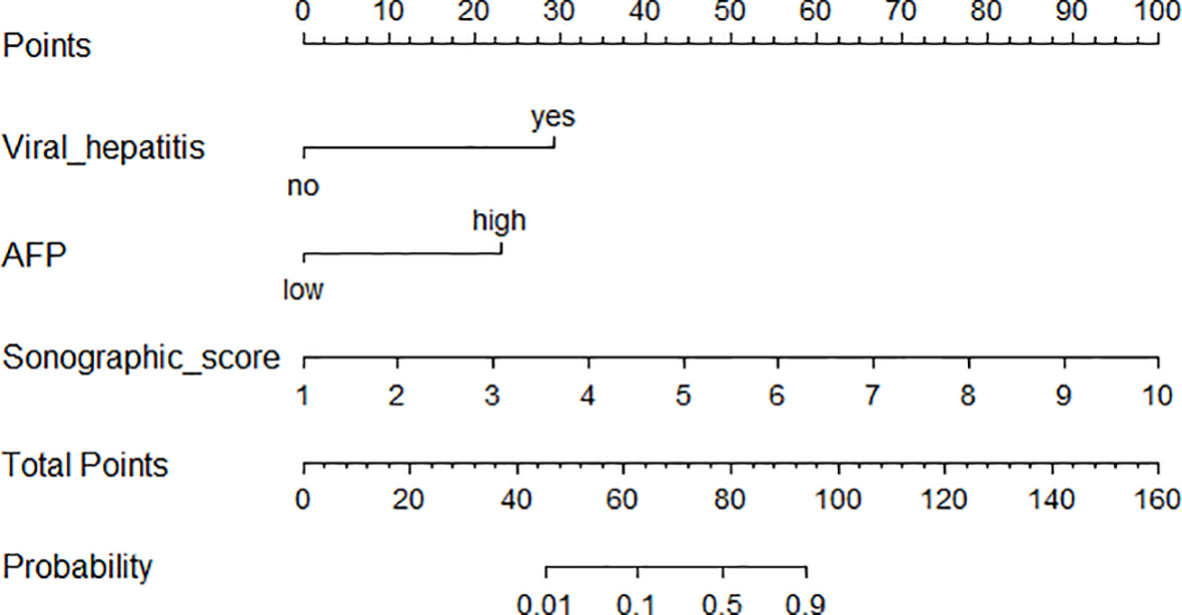
Nomogram incorporating sonographic score and clinical features for distinguishing HCC from IPT.

Based on the three independent factors from multivariate regression, a nomogram that discriminates HCC from IPT was developed. As shown in **Figure 5**, with AFP, viral hepatitis, and sonographic score, the probability of HCC could be estimated for each patient. The C-index of the nomogram was 0.991.

**Figure 6:**
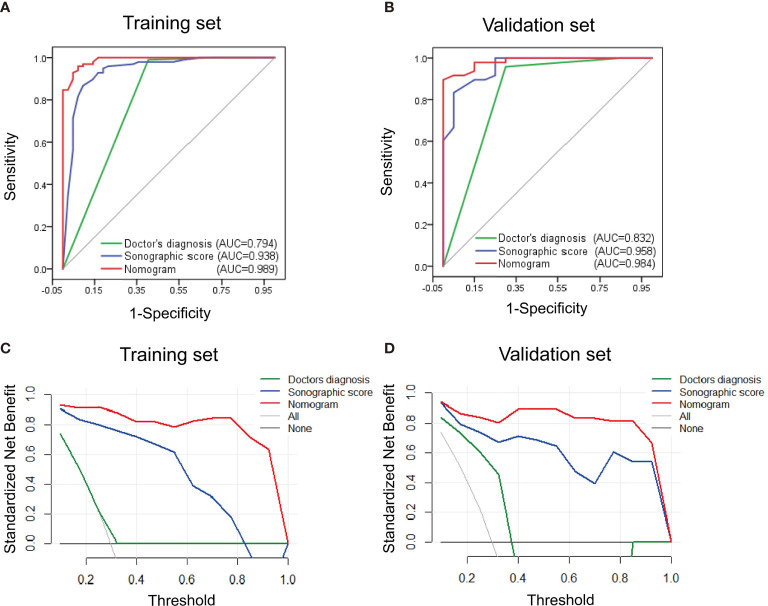
Performance and clinical usefulness evaluation of nomogram. **(A, B)** ROC curves of nomogram, sonographic score, and ultrasound doctor’s diagnosis derived from the training set **(A)** and validation set **(B)**. The AUC in both sets showed that the nomogram had a better performance than sonographic score alone and the doctor’s diagnosis. (C, D) Decision curve analysis derived from the training set **(C)** and validation set **(D)**. The y-axis measures the standardized net benefit, which is the difference between the expected benefit and the expected harm associated with each model. The black line represents the assumption that all patients were diagnosed with IPT. The gray line represents the assumption that all patients were diagnosed with HCC. The result showed that when the threshold probability was 0.1–1.0, using the nomogram added more benefit for patients than using the sonographic score alone or the doctor’s diagnosis.

The performance of nomogram was validated and compared with the sonographic score model and diagnosis made by the ultrasound doctor. ROC curves for both training and validation sets are shown in **Figures 6A, B**. The effectiveness of nomogram was better than sonographic score and the doctor’s diagnosis. The AUC for doctor’s diagnosis, sonographic score, and nomogram in the training set were 0.794, 0.938, and 0.989, respectively. The AUC for doctor’s diagnosis, sonographic score, and nomogram in the validation set were 0.832, 0.958, and 0.984, respectively. The significant differences of AUC are shown in **Supplementary Table S1**. AUCs of nomogram and sonographic score were significantly higher than that of doctor’s diagnosis. AUC of nomogram (incorporating clinical indicators and sonographic score) was higher than that of sonographic score in training set, but was not significantly different from that of sonographic score in validation set. Furthermore, clinical usefulness of the three models were compared by DCA, shown in **Figures 6C, D**. For both training and validation sets, the DCA curves showed that using the nomogram to distinguish HCC and IPT added more benefit for patients than using the other two models when the threshold probability was 0.2–1.0.

## Discussion

In this study, we developed a highly effective model incorporating CEUS and clinical features to predict the diagnosis of HCC and IPT. This is the first model that helps in the clinical practice with distinguishing between HCC and IPT.

The sonographic score consisting with only five ultrasonographic features has an AUC of over 0.93 for both training and validation sets, suggesting that the identified ultrasonographic features are of great value in the differential diagnosis. Enhancement degree during arterial phase, peritumoral vessels and liver background are positively correlated with higher possibility of HCC. Early washout and peritumoral enhancement indicate an increased probability of IPT. Hyperenhancement in arterial phase is typical in HCC (16); IPT can present with hyperenhancement, but there are also cases of iso- or hypoenhancement (23.8% and 7.1% in our study). The result suggests that hyperenhancement is significantly more common in HCC than IPT, which is consistent with previous report. Cirrhosis or diffuse parenchymal liver disease often results from chronic hepatitis infection, and HCC can develop under this kind of liver background (17–19). Whereas, IPT lesion is unlikely to be associated with certain liver background. Therefore, our results support the existing findings. Surprisingly, early washout (<60 s) indicated higher probability of IPT. Previous study showed that around 40% of enhanced IPT had quick wash-in and wash-out (6). In our study, 61.9% of IPT patients have early washout, suggesting an even earlier washout in IPT. Early washout can be present in HCC but with higher possibility than IPT, which reminds radiologists to pay attention to early washout cases for atypical diagnosis. Peritumoral enhancement is more common in IPT, probably due to the inflammatory edema zone caused by stimulatory factors or IgG4-related autoimmune reaction in IPT (20–22).

In our nomogram model, AFP and hepatitis virus infection are the clinical features that distinguish HCC from IPT. The net benefit notably increased with these clinical features added in the nomogram. AFP is a specific biomarker for HCC that has been widely used (23). Recently, Masataka et al. reported an atypical IPT with elevated AFP and AFP-L3 that mimics the characteristics of HCC (5). However, AFP is still a significant indicator for HCC in our model. Hepatitis virus infection, especially hepatitis C virus (HCV), contributes to around 50% of HCC in Western countries, while in Asian countries, hepatitis virus, mainly HBV infection, is the predominant risk factor for HCC (24–26). Furthermore, hepatitis virus infection has not been reported to be associated with hepatic IPT yet (27). Therefore, our result that hepatitis virus infection adds the possibility of HCC in the differential diagnosis was consistent with previous findings. In general, with the combination of sonographic score and clinical features, the nomogram has an AUC over 0.98 and a C-index of 0.991, indicating a superior performance as a predictive model.

In recent years, computer-aided techniques that translate high-throughput imaging information into radiomics data have been used for diagnosis (28–30). Our study, however, extracted ultrasound imaging features by artificial identification instead. On the one hand, the analysis could be less sensitive due to lack of high-throughput screening, and the imaging feature extraction could be subjective, which is a limitation of this study. Meanwhile, it suggests the application of our model does not require specific computer-aided technique, and the evaluation for the patients can be achieved with readily accessible images and clinical information, which makes our model easy to use in the practice. Furthermore, even without computer-aided techniques, our sonographic score had an excellent performance (AUC = 0.938 and 0.958 for training and validation sets, respectively), indicating our analysis with artificial imaging identification generated satisfactory results.

This study has several limitations. First, our nomogram is a binary model that only predicts the diagnosis between IPT and HCC. However, atypical IPT also mimics other diseases such as metastatic malignancy or intrahepatic cholangiocarcinoma (31, 32). To improve the accuracy and practicality, a multinomial model should be ideally developed, that differentiates IPT from all kinds of possible diagnosis. Second, the number of our cases is limited due to the rareness of IPT. Fortunately, the model works well in the validation cohort, suggesting that this model is generally reliable and has application value.

In conclusion, a sonographic signature was identified to differentiate HCC from atypical IPT preoperatively, which substantially improved the diagnostic performance compared with radiologists’ experience. A predictive nomogram combining sonographic and clinical features may potentially help with individualized diagnosis and precise medical treatment in clinical practice.

## Data Availability Statement

The raw data supporting the conclusions of this article will be made available by the authors, without undue reservation.

## Ethics Statement

The studies involving human participants were reviewed and approved by the ethics committee of Xiangya Hospital Central South University. Written informed consent to participate in this study was provided by the participants’ legal guardian/next of kin. Written informed consent was obtained from the individual(s) for the publication of any potentially identifiable images or data included in this article.

## Author Contributions

ML and CW collected and analyzed the data and drafted the manuscript. BZ, QJ, and JuL helped with data collection. JiL designed the study. All authors contributed to the article and approved the submitted version.

## Conflict of Interest

The authors declare that the research was conducted in the absence of any commercial or financial relationships that could be construed as a potential conflict of interest.

## Publisher’s Note

All claims expressed in this article are solely those of the authors and do not necessarily represent those of their affiliated organizations, or those of the publisher, the editors and the reviewers. Any product that may be evaluated in this article, or claim that may be made by its manufacturer, is not guaranteed or endorsed by the publisher.

## Supplementary Material

Supplementary Figure 1 Representative ultrasound images of IPT lesion, case 1. A male patient, aged 49, diagnosed as IPT by percutaneous liver biopsy. **(A)** Two-dimensional image showed a 2.5 × 1.8 cm nodule in the right paramedian sector (segment V) with obscure boundary, irregular shape, and heterogeneous echo distribution. **(B)** Color Doppler flow imaging (CDFI) showed sparse and dotted blood flow signal. **(C)** The nodule showed heterogeneous hyperenhancement at 18 s after 2.4 ml of SonoVue was intravenously injected *via* the antecubital vein. **(D)** Hypoenhancement at 50 s after injection.

Supplementary Figure 2 Representative ultrasound images of IPT lesion, case 2. A male patient, aged 52, diagnosed as IPT by surgical pathology results. **(A)** Two-dimensional image showed a 2.3 × 1.4 cm hypoechoic nodule in the right paramedian sector (segment VIII) with obscure boundary, oval shape and homogeneous echo distribution. **(B)** CDFI showed no blood flow signal. **(C)** The nodule showed homogeneous enhancement in arterial phase in CEUS, the area of enhancement was larger than that on two-dimension image (arrow), which was peritumoral enhancement. **(D)**. Hypo-enhancement in portal phase.

Supplementary Figure 3 Representative ultrasound images of HCC lesion. A male patient aged 60, HBsAg (+), diagnosed as well-differentiated HCC by surgical pathology results. **(A)** Two-dimension image showed a 5.4 × 4.3 cm nodule in the right paramedian sector (segment VIII) with clear boundary, regular shape, and heterogeneous echo distribution. **(B)** CDFI showed strip blood flow signal at the boundary. **(C)** Thick and curved feeding artery (arrow) in the arterial phase in CEUS. **(D)** Peritumoral vessels in the arterial phase in CEUS.
