# Supplementary figures and images for: Distinguishing Hepatocellular Carcinoma From Hepatic Inflammatory Pseudotumor Using a Nomogram Based on Contrast-Enhanced Ultrasound
Source: Front Oncol. 2021 Oct 7;11:737099. doi: 10.3389/fonc.2021.737099 (PMC8529164; doi:10.3389/fonc.2021.737099)

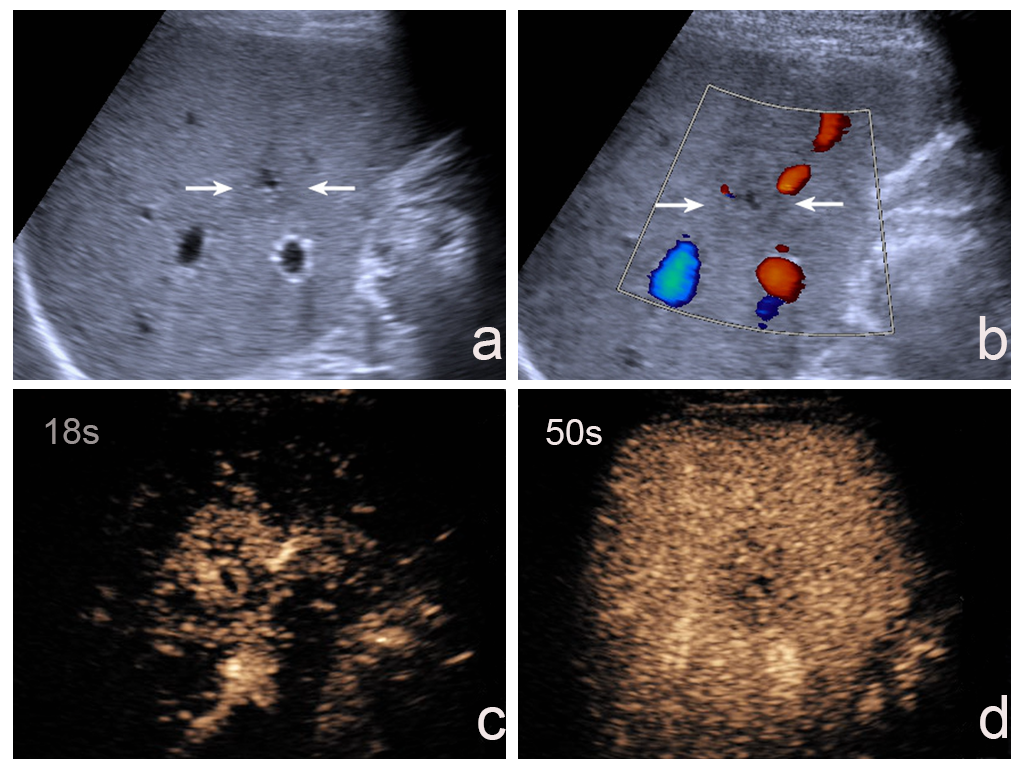

Supplement: Supplementary Figure 1 — Representative ultrasound images of IPT lesion, case 1. A male patient, aged 49, diagnosed as IPT by percutaneous liver biopsy. (A) Two-dimensional image showed a 2.5 × 1.8 cm nodule in the right paramedian sector (segment V) with obscure boundary, irregular shape, and heterogeneous echo distribution. (B) Color Doppler flow imaging (CDFI) showed sparse and dotted blood flow signal. (C) The nodule showed heterogeneous hyperenhancement at 18 s after 2.4 ml of SonoVue was intravenously injected via the antecubital vein. (D) Hypoenhancement at 50 s after injection. [file Image_1.tif]

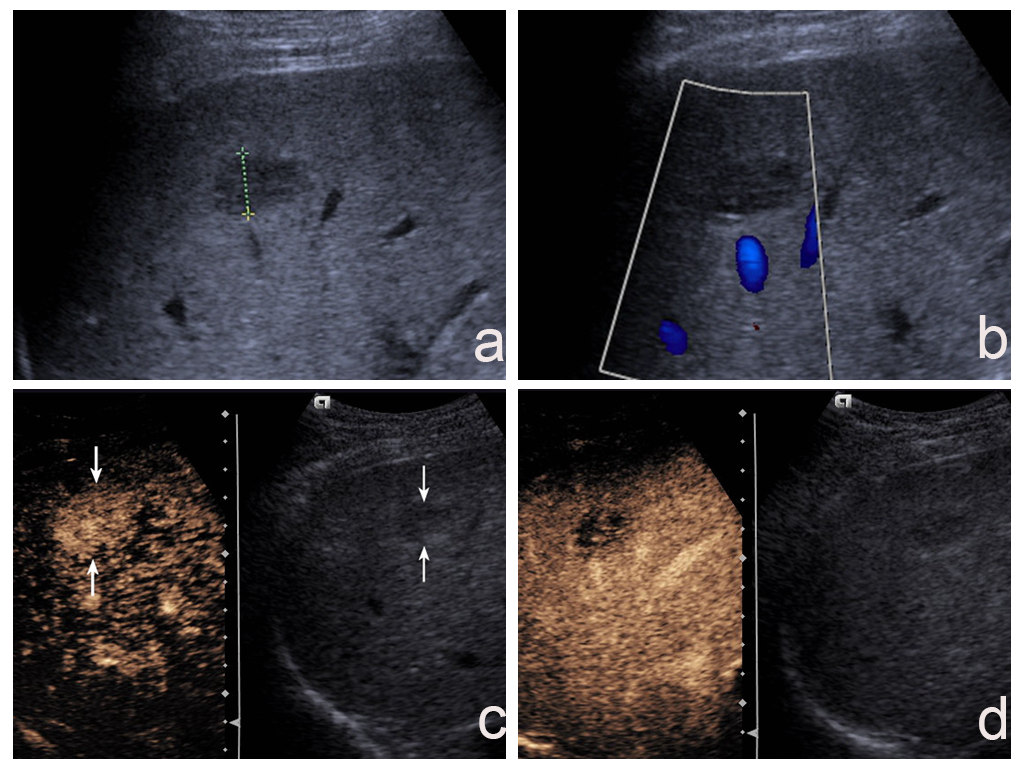

Supplement: Supplementary Figure 2 — Representative ultrasound images of IPT lesion, case 2. A male patient, aged 52, diagnosed as IPT by surgical pathology results. (A) Two-dimensional image showed a 2.3 × 1.4 cm hypoechoic nodule in the right paramedian sector (segment VIII) with obscure boundary, oval shape and homogeneous echo distribution. (B) CDFI showed no blood flow signal. (C) The nodule showed homogeneous enhancement in arterial phase in CEUS, the area of enhancement was larger than that on two-dimension image (arrow), which was peritumoral enhancement. (D). Hypo-enhancement in portal phase. [file Image_2.tiff]

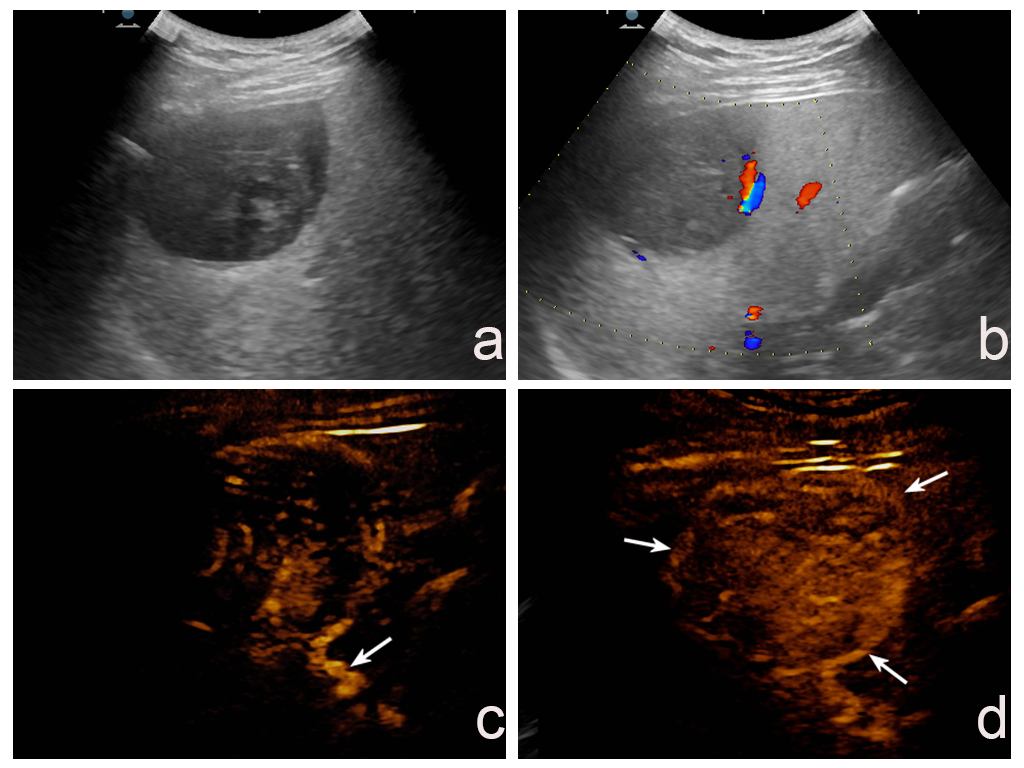

Supplement: Supplementary Figure 3 — Representative ultrasound images of HCC lesion. A male patient aged 60, HBsAg (+), diagnosed as well-differentiated HCC by surgical pathology results. (A) Two-dimension image showed a 5.4 × 4.3 cm nodule in the right paramedian sector (segment VIII) with clear boundary, regular shape, and heterogeneous echo distribution. (B) CDFI showed strip blood flow signal at the boundary. (C) Thick and curved feeding artery (arrow) in the arterial phase in CEUS. (D) Peritumoral vessels in the arterial phase in CEUS. [file Image_3.tif]
