# Supplementary material for: Distinguishing Hepatocellular Carcinoma From Hepatic Inflammatory Pseudotumor Using a Nomogram Based on Contrast-Enhanced Ultrasound
Source: Front Oncol. 2021 Oct 7;11:737099. doi: 10.3389/fonc.2021.737099 (PMC8529164; doi:10.3389/fonc.2021.737099)
Supplement: Supplementary file 4 [file Table_1.docx]

Supplementary Table 1. AUC of the nomogram, sonographic score and ultrasound (US) doctor’s diagnosis

|  | Training set (n=140) | | Validation set (n=68) | |
| --- | --- | --- | --- | --- |
|  | AUC (95%CI) | *p* value | AUC (95%CI) | *p* value |
| US doctor | 0.794 (0.698, 0.890) |  | 0.832 (0.705, 0.959) |  |
| Sonographic score | 0.938 (0.888, 0.988) |  | 0.958 (0.913, 1.000) |  |
| Nomogram | 0.989 (0.977, 1.000) |  | 0.984 (0.963, 1.000) |  |
| US doctor vs. Score |  | *0.000* |  | *0.041* |
| US doctor vs. Nomogram |  | *0.000* |  | *0.005* |
| Score vs. Nomogram |  | *0.026* |  | 0.142 |
